# Dosimetric evaluation of the skin-sparing effects of 3-dimensional conformal radiotherapy and intensity-modulated radiotherapy for left breast cancer

**DOI:** 10.18632/oncotarget.13830

**Published:** 2016-12-09

**Authors:** In Young Jo, Shin-Wook Kim, Seok Hyun Son

**Affiliations:** ^1^ Department of Radiation Oncology, Samsung Medical Center, Seoul, Korea; ^2^ Department of Radiation Oncology, Incheon St. Mary's Hospital, College of Medicine, The Catholic University of Korea, Seoul, Korea

**Keywords:** breast cancer, 3-dimensioanl conformal radiotherapy, intensity-modulated radiotherapy, radiation-related dermatitis, skin-sparing

## Abstract

The purpose of this study was to evaluate the skin-sparing effects of 3-dimensional conformal radiotherapy (3D-CRT) and intensity-modulated radiotherapy (IMRT) in patients with early left-sided breast cancer. Twenty left breast cancer patients treated with whole breast radiotherapy following breast-conserving surgery were enrolled in this study, and the 3D-CRT and IMRT plans were generated for each patient. To evaluate the dose delivered to the skin, 2 mm thickness skin (2-mm skin) and 3 mm thickness skin (3-mm skin) were contoured and a dosimetric comparison between the 2 plans was performed. The target volume coverage was better in IMRT than in 3D-CRT. The mean dose was 50.8 Gy for 3D-CRT and 51.1 Gy for IMRT. V40Gy was 99.4% for 3D-CRT and 99.9% for IMRT. In the case of skin, the mean dose was higher in 3D-CRT than in IMRT (mean dose of 2-mm skin: 32.8 Gy and 24.2 Gy; mean dose of 3-mm skin: 37.2 Gy and 27.8 Gy, for 3D-CRT and IMRT, respectively). These results indicated that the skin-sparing effect is more prominent in IMRT compared to 3D-CRT without compromising the target volume coverage.

## INTRODUCTION

Treatment for early breast cancer has changed in recent decades from radical mastectomy to breast-conserving surgery (BCS) followed by postoperative adjuvant radiotherapy (RT), which is currently the treatment of choice, having shown excellent clinical results in terms of local control and overall survival [1]. By reducing mortality from breast cancer, patients became much more aware of several treatment-related complications affecting their quality of life. Among these, commonly observed complication is radiation-related dermatitis caused by whole breast radiotherapy (WBRT).

Skin is the largest organ in the human body. Depending on the location, the skin has an average thickness of 2–3 mm in healthy adults. There are on average 650 sweat glands, 20 blood vessels, 60,000 melanocytes, and more than 1,000 nerve endings in the space of 1 square inch of skin. For this reason, most breast cancer patients who undergo BCS followed by adjuvant RT develop various degree of radiation-related dermatitis with pain and other skin toxicities [2].

Recently, intensity-modulated radiotherapy (IMRT) has been increasingly used for breast cancer. When compared to traditional 3-dimensional conformal radiotherapy (3D-CRT), IMRT enhances the target volume coverage and effectively reduces the higher dose delivered to organs at risk (OARs) such as the heart, ipsilateral lung, and so on [3]. In the case of radiation-related dermatitis, it has been reported that IMRT reduced severe acute skin toxicity according to the Radiation Therapy Oncology Group (RTOG) criteria [4]. In this study, we performed a dosimetric comparison between 3D-CRT and IMRT, and evaluated the skin-sparing effects of these 2 techniques.

## RESULTS

### Comparison of target volume coverage and dose delivered to OARs between 3D-CRT and IMRT plans

**Figure 1:**
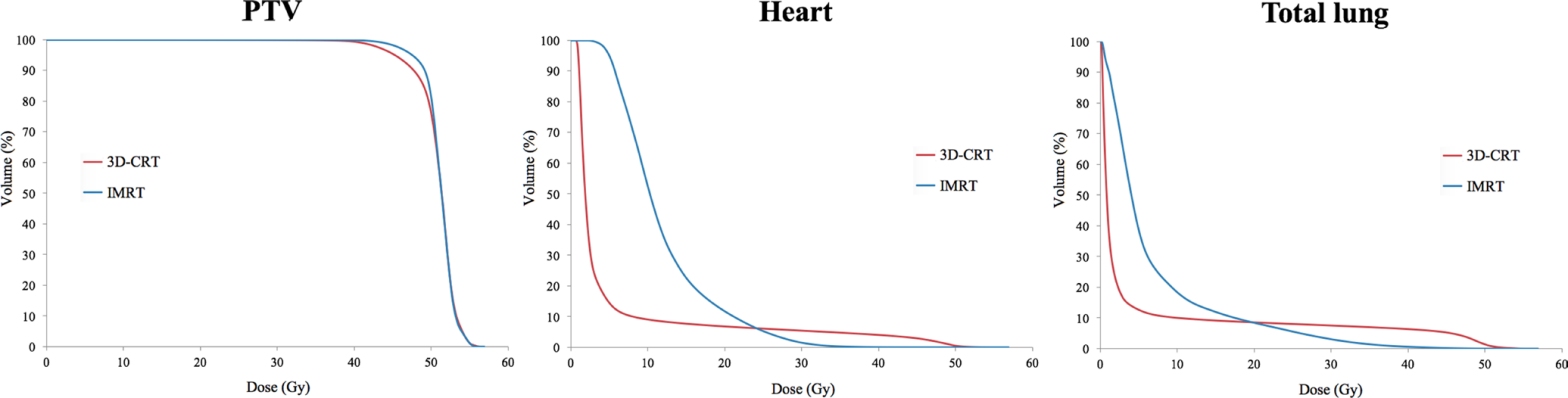
Comparison of dose-volumetric histograms for PTV, heart, and total lung between 3D-CRT and IMRT

The target volume coverage was better in IMRT than in 3D-CRT. The mean dose was 50.8 ± 1.0 Gy for 3D-CRT and 51.1 ± 1.0 Gy for IMRT. V_40Gy_ was 99.4 ± 0.8% for 3D-CRT and 99.9 ± 0.1% for IMRT. V_50Gy_ was 76.0 ± 11.5% for 3D-CRT and 80.9 ± 12.2% for IMRT. In the case of the heart, IMRT significantly reduced the dose delivered more than 30 Gy (V_30Gy_: 5.4 ± 3.0% and 1.5 ± 1.2%; V_40Gy_: 4.0 ± 2.5% and 0.0 ± 0.1%, for 3D-CRT and IMRT). However, the mean dose was increased in IMRT compared to 3D-CRT (mean dose: 5.0 ± 1.6 Gy and 11.9 ± 1.3 Gy, for 3D-CRT and IMRT, respectively). In the case of the lung, the mean dose and low-to-moderate dose was increased in IMRT compared to 3D-CRT (mean dose: 4.9 ± 1.2 Gy and 6.8 ± 7.7 Gy; V_20Gy_: 8.5 ± 2.8% and 8.4 ± 2.6%; V_30Gy_: 7.5 ± 2.6% and 3.0 ± 1.3%, for 3D-CRT and IMRT, respectively). The data are summarized in Table 1 and the cumulative DVH was shown in Figure 1.

**Table 1:** Dosimetric comparison of the dose delivered to target volume and OARs between 3D-CRT and IMRT

	Parameters	3D-CRT	IMRT	*p*-value
PTV	Mean dose (Gy)	50.8 ± 1.0	51.1 ± 1.0	< 0.001
	V_40Gy_ (%)	99.4 ± 0.8	99.9 ± 0.1	0.009
	V_50Gy_ (%)	76.0 ± 11.5	80.9 ± 12.2	< 0.001
Heart	Mean dose (Gy)	5.0 ± 1.6	11.9 ± 1.3	< 0.001
	V_30Gy_ (%)	5.4 ± 3.0	1.5 ± 1.2	< 0.001
	V_40Gy_ (%)	4.0 ± 2.5	0.0 ± 0.1	< 0.001
LV	Mean dose (Gy)	8.5 ± 3.1	13.5 ± 1.3	< 0.001
	V_30Gy_ (%)	11.2 ± 6.1	1.3 ± 1.3	< 0.001
	V_40Gy_ (%)	8.5 ± 5.3	0.0 ± 0.0	< 0.001
Ipsilateral lung	Mean dose (Gy)	10.5 ± 2.5	11.2 ± 2.2	0.321
	V_20Gy_ (%)	19.3 ± 5.6	19.1 ± 5.6	0.908
	V_30Gy_ (%)	17.0 ± 5.4	6.9 ± 2.8	< 0.001
Total lung	Mean dose (Gy)	19.3 ± 5.6	19.1 ± 5.6	0.908
	V_20Gy_ (%)	8.5 ± 2.8	8.4 ± 2.6	0.828
	V_30Gy_ (%)	7.5 ± 2.6	3.0 ± 1.3	< 0.001

Abbreviations: 3D-CRT, 3-dimensional radiotherapy; IMRT, Intensity-modulated radiotherapy; PTV, Planning target volume; LV, left ventricle; V_n_, % volume receiving > n Gy

### Comparison of skin dose between 3D-CRT and IMRT plans

**Figure 2:**
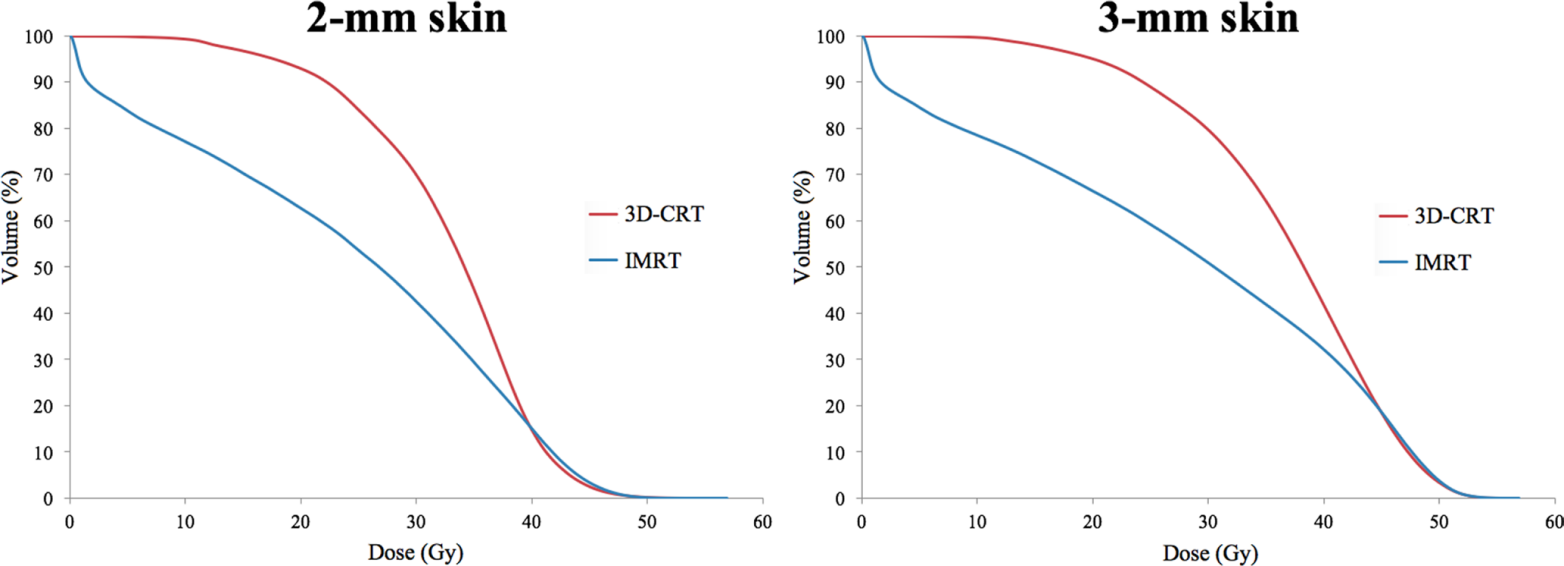
Comparison of dose-volumetric histograms for 2-mm skin and 3-mm skin between 3D-CRT and IMRT

In both 2-mm skin and 3-mm skin, the delivered dose showed a similar pattern. The mean dose and values of V_30Gy_ were higher in 3D-CRT than in IMRT (mean dose of 2-mm skin: 32.8 ± 1.4 Gy and 24.2 ± 1.6 Gy; mean dose of 3-mm skin: 37.2 ± 1.1 Gy and 27.8 ± 1.9 Gy; V_30Gy_ of 2-mm skin: 69.8 ± 5.1% and 42.5 ± 4.3%; V_30Gy_ of 3-mm skin: 79.6 ± 4.6% and 50.9 ± 4.5%, for 3D-CRT and IMRT, respectively). These results indicated that the skin-sparing effect is more prominent in IMRT. However, the value of V_40Gy_ was higher in IMRT than in 3D-CRT (V_40Gy_ of 2-mm skin: 14.7 ± 8.0% and 15.3 ± 4.3%, for 3D-CRT and IMRT), although the difference was less than 1% and statistically not significant. The data are summarized in Table 2 and the cumulative DVH is shown in Figure 2.

**Table 2:** Dosimetric comparison of the dose delivered to the skin between 3D-CRT and IMRT

	Parameters	3D-CRT	IMRT	*p*-value
2 mm-skin	Mean dose (Gy)	32.8 ± 1.4	24.2 ± 1.6	< 0.001
	V_30Gy_ (%)	69.8 ± 5.1	42.5 ± 4.3	< 0.001
	V_40Gy_ (%)	14.7 ± 8.0	15.0 ± 4.3	0.824
3 mm-skin	Mean dose (Gy)	37.2 ± 1.1	27.8 ± 1.9	< 0.001
	V_30Gy_ (%)	79.6 ± 4.6	50.9 ± 4.5	< 0.001
	V_40Gy_ (%)	41.7 ± 9.9	32.1 ± 3.6	< 0.001

Abbreviations: 3D-CRT, 3-dimensional radiotherapy; IMRT, Intensity-modulated radiotherapy; V_n_, % volume receiving > n Gy

## DISCUSSION

Radiation-related dermatitis, which frequently occurred in patients treated with WBRT, could cause mental and physical suffering due to pain, edema, and various cosmetic problem. To reduce radiation-related dermatitis, various topical agents in the irradiated breast were actively studied. However, there is still a lack of evidence and controversy surrounding this issue. For this reason, we should pay attention to skin-sparing IMRT, which is active method to reduce radiation-related dermatitis. Therefore, in this study, we compared 3D-CRT and IMRT, and evaluated the skin-sparing effect of each plan using dosimetric parameters.

Several studies have revealed that the target volume coverage was improved and the dose delivered to OARs was reduced in IMRT compared to 3D-CRT [3, 5, 6]. In this study, the higher dose delivered to the heart and the mean dose of the heart were effectively reduced, and the higher dose delivered to the lung was also reduced in IMRT than in 3D-CRT. The target volume coverage was more sufficient in IMRT than in 3D-CRT. These findings were consistent with those of previously reported studies [3, 5, 6]. Gagliardi et al. performed research on prediction of an excessive cardiac mortality in patients treated with WBRT using a normal tissue complication probability model, and reported that the cardiac mortality risk increased rapidly with more than 30 Gy [6, 7]. van Nimwegen et al. suggested linear relationship between the mean cardiac dose and rates of major coronary events, with a 7.4% increment per Gy [8]. When considering radiation-related toxicities, IMRT could provide reduced irradiation to the heart without compromising target volume coverage, which is an advantage of the technique.

Kestin et al. reported that IMRT on the breast showed no grade 3 or worse acute skin toxicity according to the RTOG toxicity criteria [4]. The study concluded that dose homogeneity across the breast in the IMRT plan can reduce the adverse effects related to the skin toxicity. Marie et al. analyzed various radiation-related factors in terms of cosmetic outcome, and found that radiation doses delivered to the entire breast and treatment volume were statistically significant factors [9]. A dose of more than 52 Gy irradiated to the entire breast showed sharp decline in the cosmetic result. Other studies also revealed that the radiation dose to the entire breast was related to the cosmetic outcome [10, 11]. However, to the best of our knowledge, there is no study comparing the skin dose using dosimetric parameters between 3D-CRT and IMRT.

In this study, the skin-sparing effect is found to be prominent in the IMRT plan compared to the 3D-CRT plan. The mean dose and V_30Gy_ of the skin was much higher in 3D-CRT than in IMRT, and DVH showed a large difference between the 2 plans (difference: 27.3% and 28.7%, for 2-mm skin and 3-mm skin, respectively). Focally, there is a short interval in which the dose delivered to the skin is reversely higher in IMRT than in 3D-CRT, but the difference is less than 1% in both 2-mm skin and 3-mm skin, and is not statistically significant. The skin-sparing effect of IMRT can vary according to the intent, purpose, or method of planning. It is possible that the dose delivered to the skin could be increased or further decreased when compared to this study. However, it should be considered that the target volume coverage should not be compromised solely with the intent of decreasing the dose to the skin. The results of this study are meaningful because the target volume coverage of IMRT is improved compared to 3D-CRT and the skin dose also decreased.

In conclusion, IMRT can effectively reduce the dose delivered to OARs as well as adequately improve the target volume coverage. In terms of skin-sparing, IMRT can also reduce the skin dose, and therefore reduce the risk of radiation-related dermatitis.

## MATERIALS AND METHODS

### Patients, simulation, and target delineation

Left breast cancer patients who visited our institution from November 2014 to April 2015 were included in this study. Among them, 20 patients were randomly selected and further investigated. Before simulation computed tomography (CT), whole breast tissue of each patient was wired using radio-opaque material. Simulation CT was performed using a LightSpeed RT16 CT scanner (GE Healthcare, Waukesha, WI) with 2.5 mm thickness. When simulation CT was conducted, all patients used a vac-lock immobilization device with a 10 degree tilted breast board. Eclipse version 8.9 (Varian Medical Systems, Palo Alto, CA) was used as a radiation treatment planning system.

The planning target volume (PTV) of the left breast, heart, left ventricle, both lung, and spinal cord were delineated. To evaluate the dose delivered to the skin, 2 mm thickness skin (2-mm skin) and 3 mm thickness skin (3-mm skin) were also contoured. The average volume of PTV was 453.6 cm^3^ (range: 180.1 to 761.4 cm^3^). For the consistency of target volume and OARs, all of these were contoured by a single experienced radiation oncologist. The PTV was based on the RTOG atlas [12]. However, the PTV was edited according to the wired area, surgical clip, and seroma, and we trimmed the anterior border by 3 mm from the skin for skin-sparing treatment planning.

### Treatment planning for 3D-CRT and IMRT

For the 3D-CRT plan, to remove unexpected hot spots and improve homogeneity for PTV, we used the field-in-field technique in addition to the 2 parallel-opposed tangential fields technique that is commonly used for the treatment of breast cancer. The prescribed dose was 50 Gy in 25 fractions. A 6-MV photon beam was used and the calculation grid was 2.5 × 2.5 mm. An analytical anisotropic algorithm (version 8.9.17) was used for dose calculation. The upper margin of the main field was either 0.5 cm above the sternal notch or 2 cm above the PTV, and the lower margin was 2 cm below the inframammary fold. The medial border was the midsternum and the lateral border was 2 cm beyond palpable breast tissue (midaxillay line).

For the IMRT plan, fixed-beam IMRT with 7 fields was used. Although the angles of each field could vary individually, the intervals were the same in all patients (the beam arrangement intervals from the medial to the lateral beam: 45, 30, 20, 20, 30, and 45°). The energy, calculation grid, and algorithm were the same as those of 3D-CRT. The plans were optimized to deliver at least 95% of the prescribed dose to 95% of the PTV. In addition, we tried to reduce the radiation dose to the heart or lung as much as possible while delivering an adequate dose to the PTV.

### Statistical analysis

For this analysis, the mean dose and V_n_ (% of volume receiving > n Gy) were used as dosimetric parameters. Wilcoxon signed-rank test, a non-parametric statistics for paired *t-test*, was used for the comparison of the 2 plans. A *p-value* of less than 0.05 was considered statistically significant.
